# Book Review: The Value of Shame – Exploring a Health Resource in Cultural Contexts

**DOI:** 10.3389/fpsyg.2021.629339

**Published:** 2021-01-28

**Authors:** Wolf Axel Langewitz

**Affiliations:** ^1^University Hospital of Basel, Basel, Switzerland; ^2^University of Basel, Basel, Switzerland

**Keywords:** shame, culture, existential psychology, personal growth, pride

## Summary of the Book

The book contains eleven articles focusing on shame from different perspectives that are grouped according to “Theoretical perspectives on shame and culture,” “Culture-specific perspectives on shame,” and “The application of shame and culture in therapeutic and counseling practices.” An extensive introductory chapter by the two editors precedes these single articles. The editors summarize different perspectives on shame, present their valid arguments for the specific relevance of the discourse on shame in the context of culture, and offer interesting reflections on the potential role of shame for growth in individuals and for growth in societies. Shame is described as a potent regulatory mechanism in human interactions that helps avoid doings that harm others and finally leads to guilt and guilt-feelings. Especially in the context of culture and shame articles report on the lack of a society's willingness to reflect e.g., on the reckless behavior toward the native populations in Northern America and Australia. The authors provide disturbing facts and argue that instead of shame, a sense of pride about the achievements of the white man is preserved including his efficiency in killing “the others.” A more open stance toward a society's ability to work on guilt feelings and shame is presented in a chapter on shame in the context of Higher education in South Africa; others mention the development of a “shame identity” in Germany after 1945. In the last two articles, the authors consider the potential of shame in a therapeutic context in more detail, one of which summarizes a Jungian approach to shame, the other focussing on therapies that emphasize the need for self-forgiveness and present different approaches in this realm.

## Evaluation of the Book's Content

Being ashamed is an extremely important phenomenon in humans because it is probably the single most relevant experience of the lived body that brings together an irreducible sense of oneself, leaving no doubt that “this is me and only me in this very moment” and an irresistible stimulus to reflect on oneself. Shame is described as the emotional counterpart to pride. An interesting point here relates to the question, to what extent the perception of a change in a bodily state (“body” referred to as the “lived body” [DER LEIB] in German) is a necessary element of an “emotion.” Pride, for example, might be viewed primarily as a certain (upright) posture, whereas being proud of something could reflect the fulfillment of some self-defined tasks with less prominent “bodily” perceptions. Shame results from a transgression of norms that often reflect societal rules that possess authority and have become internalized by the individual. If culture is understood as a complex system of norms including certain language-specific opportunities to address—or avoid—shame-relevant issues, the focus on shame in the context of culture as presented in the book is more than welcome. The scope of the book is broad, the articles are very well-written, providing both a clear structure and an up-dated list of references. The editors are to be congratulated that such an ambitious undertaking resulted in a consistent and coherent book. Principally, this can be achieved in two ways: either by providing one unifying theory of the nature and development of shame to which the other articles refer, or by inviting authors to provide their own definition of shame that best suits the specific content of their chapters. The editors chose the latter variant but provide an extensive introductory chapter that helps the reader find his path through the different applications of shame in the context of culture. A positive outcome of this approach is the fact that single articles stand on their own, without reference to other chapters. A potential shortcoming is the somewhat difficult orientation within the book, which could be improved by providing a list of keywords in a future edition of the book.

## Discussion

At present, we are aware of politicians presenting faulty facts or wrong accusations, who do not mind when their lies are exposed in public; they do not show any indication of shame or remorse. If these politicians serve as role models for appropriate behavior, they pave the way toward a shame-less society, in which anything goes as long as it serves an individual's purpose. The potential of shame as a powerful motivator to change one's behavior is lost. In my view, the content of this textbook provides conclusive evidence that the development of shame is a deeply human capacity and that the sense of being ashamed is a prerequisite for personal and societal growth.

## Author Contributions

WL wrote the manuscript.

## Conflict of Interest

The author declares that the research was conducted in the absence of any commercial or financial relationships that could be construed as a potential conflict of interest.

